# Poor Biological Factors and Prognosis of Interval Breast Cancers: Long-Term Results of Bahçeşehir (Istanbul) Breast Cancer Screening Project in Turkey

**DOI:** 10.1200/GO.20.00145

**Published:** 2020-07-17

**Authors:** Neslihan Cabioğlu, Sibel Özkan Gürdal, Arda Kayhan, Nilüfer Özaydın, Cennet Şahin, Ömür Can, Beyza Özçınar, Gönül Aykuter, Gülçin Vatandaş, Erkin Aribal, Vahit Özmen

**Affiliations:** ^1^ Department of Surgery, Istanbul University, Istanbul Medical Faculty, Istanbul, Turkey; ^2^ Department of Surgery, Namık Kemal University, Faculty of Medicine, Tekirdag, Turkey; ^3^ Department of Radiology, Erzincan Binali Yıldırım University Faculty of Medicine, Erzincan, Turkey; ^4^ Department of Public Health, Marmara University, Faculty of Medicine, Istanbul, Turkey; ^5^ Department of Radiology, Şişli Etfal Research and Teaching Hospital, Istanbul, Turkey; ^6^ MEMEDER Screening Center, Bahçeşehir, Istanbul, Turkey; ^7^ Department of Radiology, Acıbadem University, Faculty of Medicine, Istanbul, Turkey

## Abstract

**PURPOSE:**

The Turkish Bahçeşehir Breast Cancer Screening Project was a 10-year, organized, population-based screening program carried out in Bahçeşehir county, Istanbul. Our aim was to examine the biologic features and outcome of screen-detected and interval breast cancers during the 10-year study period.

**METHODS:**

Between 2009 and 2019, 2-view mammograms were obtained at 2-year intervals for women aged 40 to 69 years. Clinicopathological characteristics including ER, PR, HER2-neu, and Ki-67 status were analyzed for those diagnosed with breast cancer.

**RESULTS:**

In 8,758 screened women, 131 breast cancers (1.5%) were detected. The majority of patients (82.3%) had prognostic stage 0-I disease. Contrarily, patients with interval cancers (n = 15; 11.4%) were more likely to have a worse prognostic stage (II-IV disease; odds ratio [OR], 3.59, 95% CI, 0.9 to 14.5) and high Ki-67 scores (OR, 3.14; 95% CI, 0.9 to 11.2). Interval cancers detected within 1 year were more likely to have a luminal B (57.1% *v* 31.9%) and triple-negative (14.3% *v* 1%) subtype and less likely to have a luminal A subtype (28.6% *v* 61.5%; *P* = .04). Patients with interval cancers had a poor outcome in 10-year disease-specific (DSS) and disease-free survival (DFS) compared with those with screen-detected cancers (DSS: 68.2% *v* 98.1%, *P* = .002; DFS: 78.6% *v* 96.5%, *P* = .011).

**CONCLUSION:**

Our findings suggest the majority of screen-detected breast cancers exhibited a luminal A subtype profile with an excellent prognosis. However, interval cancers were more likely to have aggressive subtypes such as luminal B subtype or triple-negative cancers associated with a poor prognosis requiring other preventive strategies.

## INTRODUCTION

Meta-analyses of randomized studies have demonstrated that mammography screening decreased breast cancer mortality in women aged 39 to 59 years and age 60 to 69 years by approximately 15% and 32%, respectively.^1^ A recent meta-analysis of 60 studies in Europe also reported a mortality reduction ranging between 33% and 43% for Northern Europe, 43% and 45% for Southern Europe, and between 12% and 58% for Western Europe.^2^ In recent years, there has been much debate on the benefit of mammographic screening related with overdiagnosis and overtreatment.^3-6^ Screen detection was associated with increased disease-specific survival (DSS) compared with symptom-detected breast cancer, independent of early stage and favorable prognostic clinicopathological factors.^7-12^ This stage-adjusted reduction in breast cancer mortality has been partially attributed to higher detection rates on screening of slow-growing, indolent tumors with low metastatic potential that would never cause symptoms or death.^13^

**CONTEXT**

**Key Objective**
The prognosis and distinguishing pathologic features of interval cancers were investigated to outline their different characteristics in the first, organized, population-based screening program in Istanbul, Turkey.
**Knowledge Generated**
In 8,758 women screened after age 40 years biannually, the majority of the 131 cancers were detected at prognostic stage 0-1. Patients with interval cancers (11%) presented with a more advanced stage, poor prognosis, and higher Ki-67 scores.
**Relevance**
Interval cancers of more aggressive subtypes and with a poor prognosis may require other early detection and prevention strategies.

Screen-detected cancers are more often smaller tumors that are lymph node negative, estrogen receptor (ER)-positive, and of low grade, compared with interval cancers.^14-16^ Studies using the prognostic molecular subtypes defined by expression profiling (ie, luminal, HER2-positive, and basal) have shown that screen-detected tumors are more likely to be luminal A subtype and less likely to be basal-like, consistent with improved outcomes.^12^

In Turkey, as a developing country bridging eastern Europe and the Middle East region, there are currently no nationwide, organized, population-based mammographic screening programs, and mostly opportunistic screening has been performed.^17,18^ the Turkish Bahçeşehir Breast Cancer Screening Project is a 10-year, organized,population based screening program (between 2009 and 2019) carried out in women aged 40 to 69 years who live in one of the largest counties of Istanbul, Turkey. The study closed at the end of 2019.^19,20^ The aim of our study was to examine the clinicopathological and biologic features of screen-detected and interval tumors among breast cancers detected during the 10-year study in the present Turkish breast screening program and determine its effect on breast cancer stage shift.

## METHODS

### Study Population

All patients with breast cancer detected during the Bahçeşehir Breast Cancer Screening Project were included in the study. Screening was performed for 8,758 women registered to the Bahçeşehir Breast Cancer Screening Center between January 2009 and January 2019 every 2 years in 5 to 10 sequential rounds. However, women with a family history of breast cancer underwent annual mammographic screening. Approval of the Institutional Review Board of Istanbul University was obtained. National Health Authorities were informed and approval was obtained. Each eligible woman signed a written informed consent.

### Screening Procedure

Data from the first invitation to screening was defined as the starting point. Two views, (mediolateral oblique), and craniocaudal, of each breast were obtained. All examinations were double read by 2 independent radiologists who were blinded to each other’s interpretations. The discordant cases were also evaluated by a third experienced breast radiologist for the definitive final decision. Mammographic findings and breast density were classified according to Breast Imaging Reporting and Data System (BI-RADS) of the American College of Radiology.^21^

Women with a mammogram classified as BI-RADS 0 were recalled for additional imaging workup, including spot compression and magnification views, ultrasonography, or magnetic resonance imaging (MRI). In case of suspicious abnormality or one highly suggestive of malignancy (BI-RADS 4 or 5 cases) in the final report, the radiologists decided on whether to proceed to an additional workup such as core needle aspiration biopsy guided by ultrasonography (14-16 gauge), or a vacuum-assisted, large-core (9-10 gauge) stereotactic biopsy. The diagnostic process was completed within 4 weeks to minimize the period of uncertainty. Patients with a diagnosis of cancer were referred to university hospitals for treatment, including surgery, chemotherapy, hormone therapy, and radiotherapy. Patients underwent regular follow-up every 3 to 6 months for 5 years after completion of their therapies, and were seen yearly after 5 years after diagnosis.

According to the European Union Breast Cancer Screening Quality Guidelines, screen-detected cancers were defined as breast cancers that were mammographically detected in the first or a subsequent screening rounds. Interval cancer is defined as symptomatic cancer diagnosed within 24 months of a negative screening by mammography with or without additional assessment, including ultrasonography in selected cases. Furthermore, missed cancers were defined as the diagnosis of cancer after a false-negative mammogram.

### Clinicopathological Characteristics

The following clinical and pathologic factors, along with intrinsic subtypes of breast cancer (ie, luminal A, luminal B, nonluminal HER2, and triple negative), were analyzed: age, tumor localization, the histopathological type, histologic grade, lymphovascular invasion, nodal status, multifocality, and ER, progesterone receptor (PR), and HER2-neu overexpression. ER or PR positivity was considered positive in any nuclear staining ≥ 1%. HER2-neu overexpression was considered either by immunohistochemistry 3+ or fluorescent in situ hybridization or silver-enhanced in situ hybridization positivity. The proliferative index Ki-67 was defined as the percentage of immunoreactive tumor cells of the total number of cells counted. The intrinsic subtypes of the tumor were defined as follows and as revised recently^22,23^: luminal A: ER+ or PR+, HER2-neu (−), Ki-67 < 20% (low proliferative activity); luminal B: ER+ or PR+, HER2-neu (+), Ki-67 ≥ 20% (high proliferative activity); nonluminal: HER2+, ER−, PR−, HER2-neu (+); triple negative: ER−, PR−, HER2-neu (−).

The *AJCC Cancer Staging Manual, 8th edition*, was used in the staging of patients considering the ER, PR, and HER2-neu expressions of tumors to determine the prognostic stage of patients’ disease, in additional to anatomic stage.^24^

### Statistical Analysis

To assess the associations between the documented variables and interval cancer status, each parameter was tested by using the Fisher exact test or χ^2^ test in 2-tailed univariate analyses. To explore the clinicopathological characteristics of true interval cancers, the radiologically missed cancers (n = 9) were excluded from the study cohort in categorical statistical analysis. Independent variables included the various clinicopathologic variables. In addition, binary logistic regression analysis was used to assess the significant associations associated with interval cancer detection rate. The dependent variable was the interval cancer detection rate. Results were reported as odds ratio (OR) with 95% CIs.

Furthermore, Kaplan-Meier survival analyses were performed to examine the disease-free survival (DFS) and DSS rates to determine the factors associated with prognosis in patients with invasive breast cancer. Log-rank test was used to analyze the difference between 2 groups to compare the prognostic effect of different variables. Radiologically missed cancers were excluded from the study cohort in log-rank test analyses to explore the prognostic effect of true interval cancer compared with other prognostic factors, including clinicopathological factors. Cox regression analyses were used to assess the hazard ratio of factors associated with prognosis. A *P* ≤ 0.05 was considered statistically significant. The SPSS, version 17.0 (IBM, Armonk, NY) was used in statistical analyses.

## RESULTS

A total of 131 breast cancers (1.5%) were detected. The median patient age was 52 (range, 40-73) years. Of the 131 patients with breast cancer, 52 (39.7%) were younger than 50 years, and the remaining 79 patients were older than 50 years. Of 131 cancers, 15 (11.5%) were true interval cancers that were detected after a negative screening by digital mammography within 24 (range, 0-23) months; 9 patients had radiologically missed cancers. Of 15 interval cancers, the majority (n = 9; 60%) were detected within 1 year (range, 0-11 months) after a negative biennial screening.

No significant difference was found between screen-detected and interval cancers with regard to demographic features, including age younger than 50 years, menopausal status, family history, use of hormone replacement therapy, and body mass index (Table 1). Screen-detected cancers were more likely to be found in the initial screening rounds, whereas interval cancers were more likely to be detected in the subsequent rounds, even though this difference did not reach statistical significance (47.7% *v* 26.3%, respectively; *P* =. 169; Table 1).

**TABLE 1:**
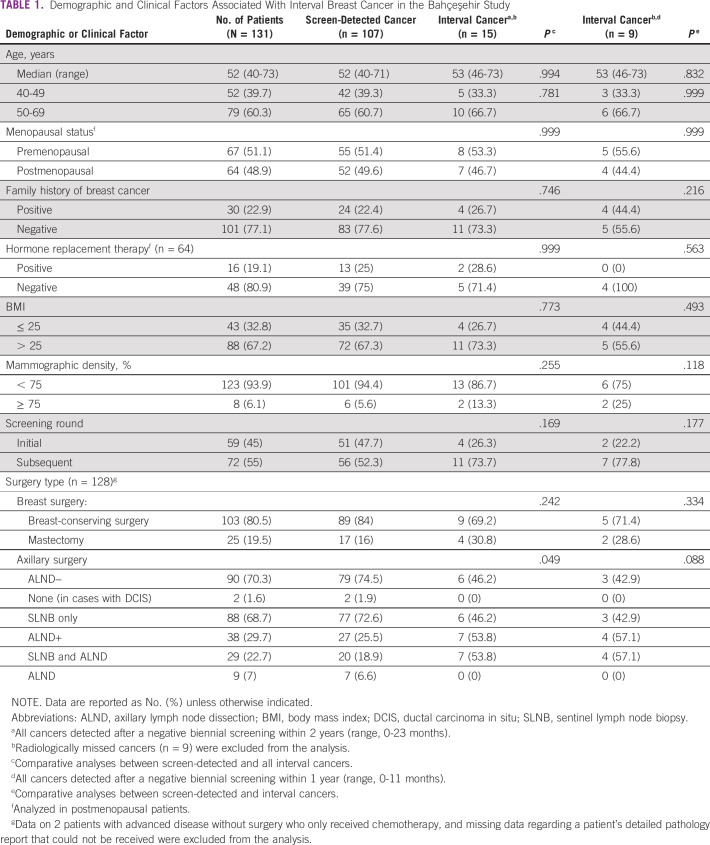
Demographic and Clinical Factors Associated With Interval Breast Cancer in the Bahçeşehir Study

Of 128 patients who underwent surgery, the majority (n = 103; 80.5%) underwent breast conservation with or without sentinel lymph node biopsy (SLNB), whereas only 38 patients (29.7%) had axillary lymph node dissection (ALND) with or without SLNB the axillary procedure (Table 1). Furthermore, patients with interval cancers were more likely to undergo axillary dissection when compared with the screen-detected group (ALND in screen-detected group: 25.5% *v* ALND in interval cancer group: 53.8%; *P* = .049), which may be due to the increased initial clinical and/or pathologic presentation with axillary lymph node positivity (Table 2). Even though there was a trend for an increase in breast-conserving surgery rate in the screen-detected group, no significant difference was found in terms of breast operation type between the 2 groups.

**TABLE 2:**
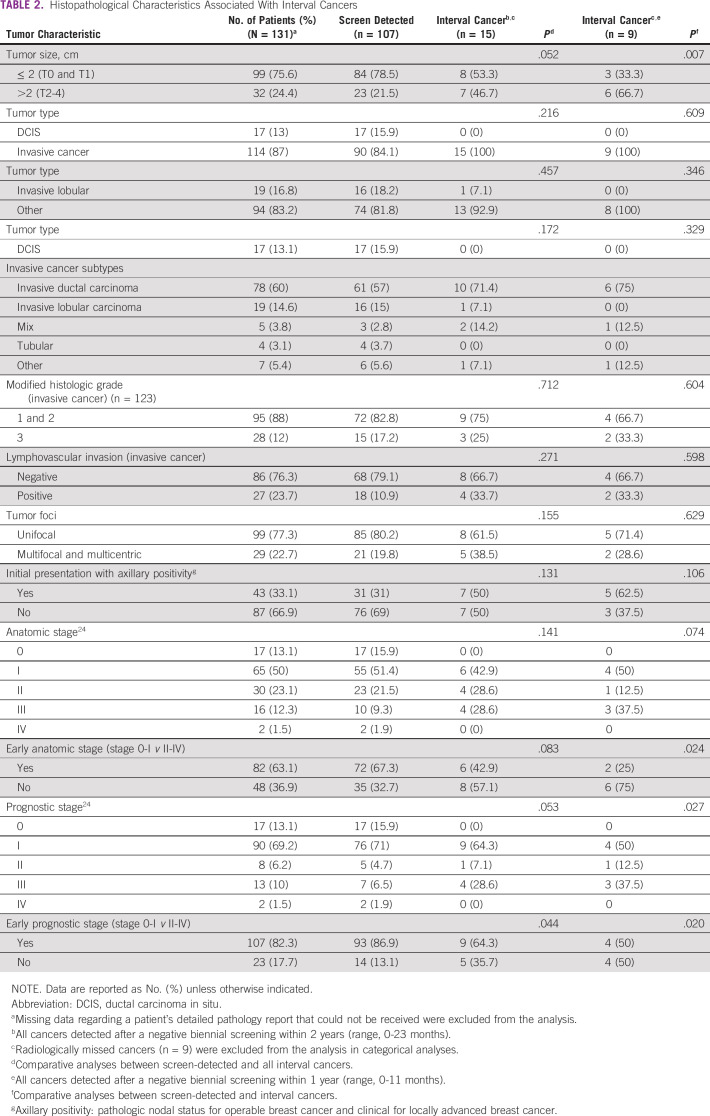
Histopathological Characteristics Associated With Interval Cancers

Of 131 tumors, 114 (87%) were invasive cancers and 17 (13%) were ductal carcinoma in situ (DCIS). All DCIS tumors were detected in patients with screen-detected cancers (Table 2). Although there was an increased trend for a histopathology having an invasive ductal component, lymphovascular invasion, and multifocality and multicentricity in the interval cancers, compared with screen-detected cancers, there was no statistical significance between the groups (Table 2). The majority of patients with screen-detected cancers (87%) had prognostic stage 0-1, whereas 64.3% of patients with interval cancers had an early prognostic stage. Patients with screen-detected cancers were more likely to present with a tumor in an early anatomic stage and prognostic stage,^24^ whereas interval cancers presented in more advanced stages, as expected (Table 2).

Among invasive cancers, 91.4% were ER+ or PR+, whereas 15% were HER2-neu (+) and 64.5% had low Ki-67 levels (< 20%). In terms of molecular subtypes, most of the invasive cancers were either luminal A (59.5%) or luminal B (31.5%), whereas other nonluminal HER2 (6.3%) and triple-negative cancers (2.7%) were detected less frequently (Table 3). Patients with interval cancers detected within 11 months of a negative screening were more likely to have high mammographic density, high Ki-67 positivity, and luminal B tumors, and less likely to have luminal A tumors, even though these associations did not reach statistical significance (Tables 1 and 3).

**TABLE 3:**
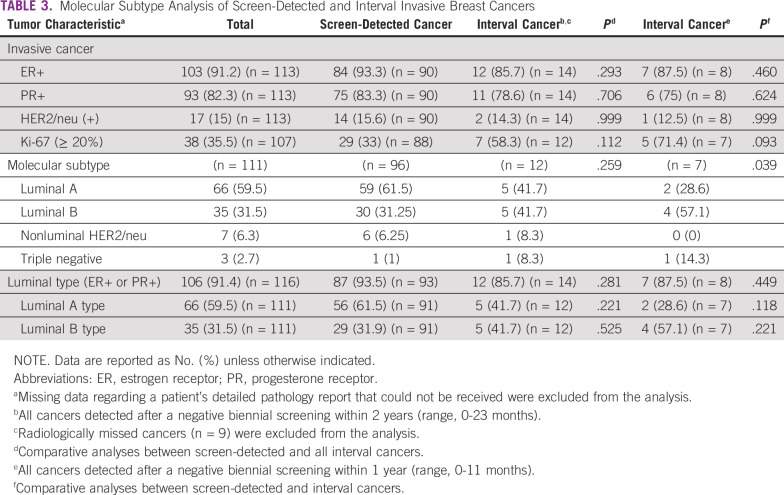
Molecular Subtype Analysis of Screen-Detected and Interval Invasive Breast Cancers

In logistic regression analysis, interval cancers were associated with more-advanced prognostic stages (prognostic stage II-IV *v* stage 0 and I; OR, 3.59; 95% CI, 0.9 to 14.5) and high Ki-67 positivity (Ki-67 ≥ 20% *v* < 20; OR, 3.14; 95% CI, 0.9 to 11.2) compared with patients with screen-detected cancers.

### Outcome

**FIG 1:**
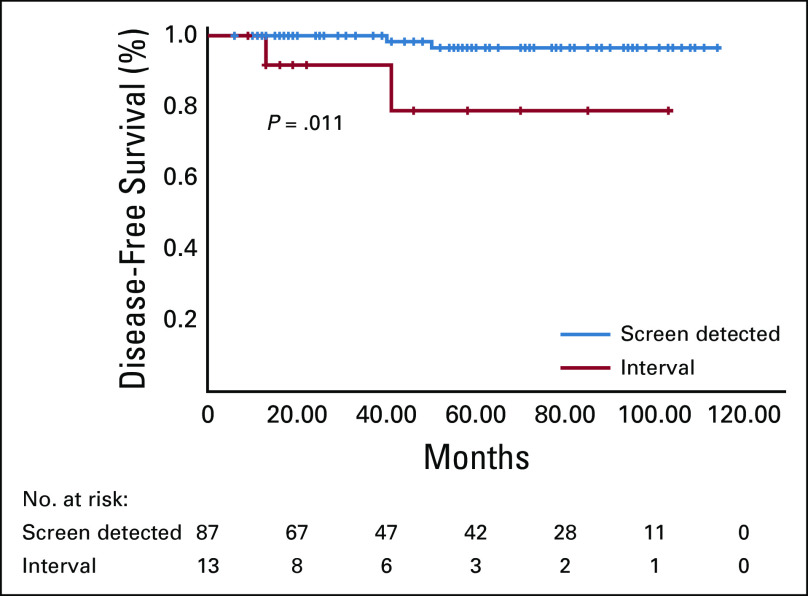
Among patients with invasive cancer, those with interval cancers had a shorter disease-free survival compared with those with screen-detected cancers (interval cancer, 78.6% *v* screen-detected cancer, 96.5%; *P* = .011). Radiologically missed cancers (n = 9) and missing data (n = 5) were excluded from the analysis.

**FIG 2:**
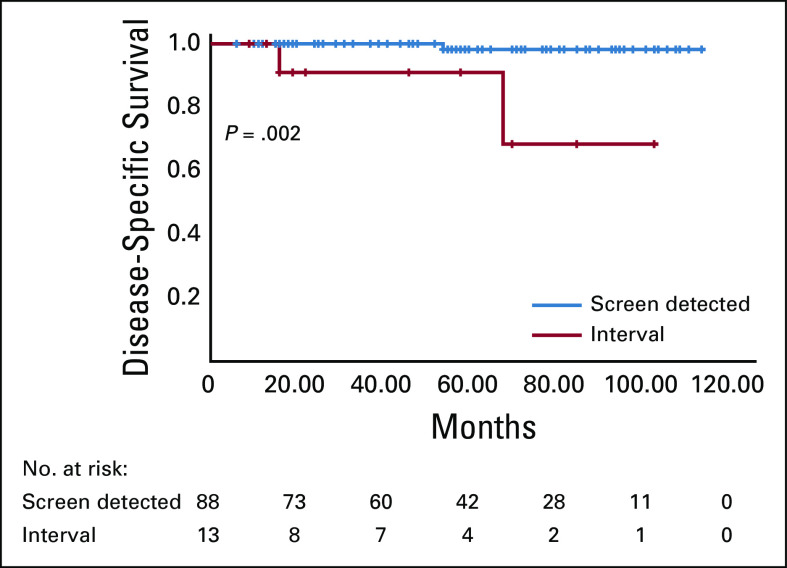
Among patients with invasive cancer, those with interval cancers had a shorter disease-specific survival compared with those with screen-detected cancers (interval cancer, 68.2% *v* screen-detected cancer, 98.1%; *P* = .002). Radiologically missed cancers (n = 9) and missing data (n = 4) were excluded from the analysis.

Median follow-up was 58 (range, 12-124) months. Of all cases (n = 131), 10-year DFS and DSS rates were 94.6% and 96%, respectively. None of the patients with DCIS had a recurrence; the 5-year DSS and DFS rates were both 100%. However, the 10-year DSS and DFS rates were 92.4% and 93.5%, respectively, for patients with invasive cancer. Patients with interval cancers (Fig 1) with a initial axillary positivity, tumor > 2 cm, multifocal and multicentric tumors, and with a more-advanced prognostic stage (ie, stage II-IV) were more likely to have a poorer DFS compared with other patients (Table 4). Similarly, patients with interval cancers (Fig 2) with a initial axillary positivity and luminal A tumors were more likely to have a worse DSS compared with other patients (Table 4). Among patients with prognostic stage I, those with interval cancers were more likely to have a worse 10-year DFS and DSS compared with other patients with screen-detected cancers.

**TABLE 4:**
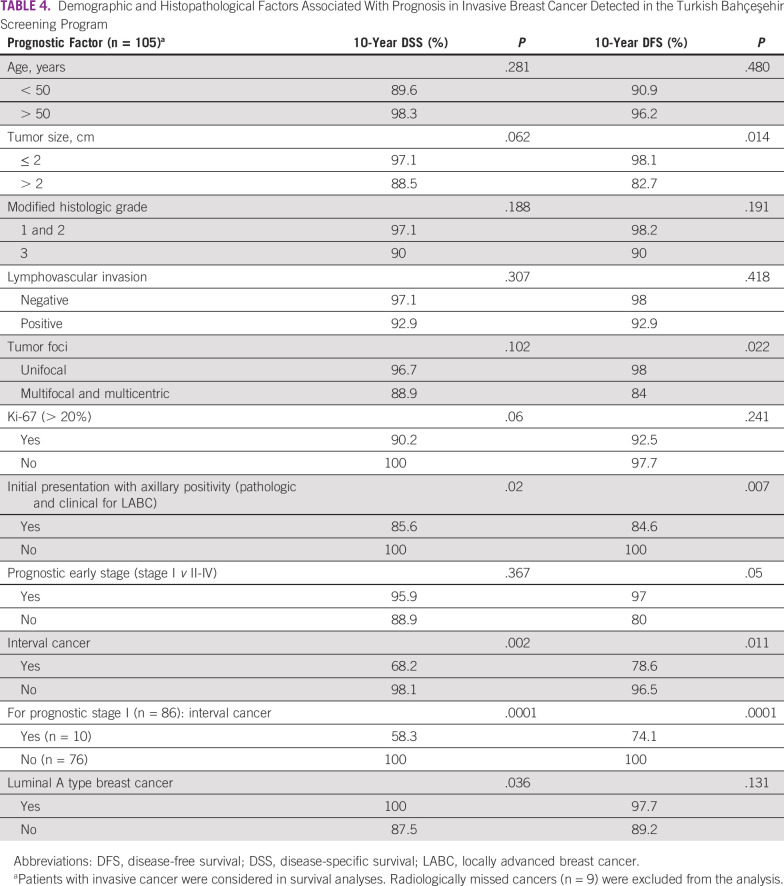
Demographic and Histopathological Factors Associated With Prognosis in Invasive Breast Cancer Detected in the Turkish Bahçeşehir Screening Program

## DISCUSSION

In recent years, the effectiveness of breast cancer screening has been strongly debated with regard to overdiagnosis and overtreatment.^3^ Many studies have found that screen-detected cancers have more favorable clinicopathologic factors and prognosis that might be associated with a different tumor biology compared with interval cancers.^8-16,25-36^ Patients with a screen-detected cancer were more likely to have a low- or even an ultra–low-risk tumor compared with interval cancers assessed by the 70-gene signature.^25,26^ Specific copy number imbalances were also noted in screen-detected breast cancers associated with more favorable, indolent tumor genotypes and might contribute to the survival advantage associated with screening.^27^ Therefore, we investigated the molec-ular subtypes and clinicopathological characteristics and prognosis of cancers detected in our organized, population-based Bahçeşehir Screening Project.

Breast cancer subtypes were identified by microarrays and immunohistochemistry and identified as luminal A, luminal B, nonluminal HER2-neu, and triple-negative tumors.^22,23^ Of those, luminal A tumors were associated with the most favorable clinical outcome. Previous studies demonstrated that the distribution of the molecular subtypes differed in screen-detected breast cancer compared with symptomatic cancers found outside of mammographic screening. Sihto et al^12^ reported that in the age group 50 to 69 years old, luminal type A was more common (73.3%) among screen-detected cancers compared with 63.8% of cancers found outside mammography screening, whereas the HER2+/ER− type was rare (5.7%) among screen-detected cancers. Crispo et al^28^ also reported increased detection rate of luminal A type cancers among screen-detected breast cancers compared with symptomatic ones. A significantly higher proportion of cases expressed PgR and had a Ki67 ≤ 20% among screen-detected cancers compared with symptomatic tumors (78.1% *v* 68%, *P* = .04; and 57.1% *v* 44.1%, *P* = .02, respectively). The majority of patients (64.5%) in our study had low Ki-67 scores (< 20%), consistent with the findings from the Sihto et al study,^12^ along with other reports.^28-30^ In an analysis of > 13,000 patients with breast cancer in the Turkish Federation of Breast Diseases Society Breast Cancer Registry, the luminal A molecular subtype was 62%, the luminal B was 15%, HER-2 neu was 8.5%, and triple-negative breast cancers were 15% in those patients with disease detected outside screening programs.^18^ In concordance with these studies, our results also show that the majority of the cancers detected in screening are luminal (91.4%) or even luminal A molecular subtype (59.5%), which is biologically the most favorable low-risk breast cancer, whereas the nonluminal HER2-neu subtype and triple-negative breast cancers were relatively rarely detected in our screening program. Furthermore, there was a trend toward an increased rate of triple-negative and luminal B type cancers and decreased rate of luminal A type tumors among interval cancers, compared with screen-detected group, consistent with reports from previous studies.^29-32^

Mammographic screening detects breast cancer at an early stage associated with improved survival rates.^16,33^ In the recent analysis of the Turkish Federation of Breast Diseases Society Breast Cancer Registry, the rates of DCIS and stage I breast cancer at diagnosis were 4.7% and 27.5%, respectively.^37^ However, in our study, we detected at least a 2.5-fold increased rate in DCIS (13.1%) and almost a 2-fold increased rate of stage I breast cancer (50%) during our mammographic screening program. In 2017, the eighth revised edition of the TNM system was introduced, incorporating the prognostic-stage tables in addition to the traditional anatomic-stage tables by considering biomarker expressions such as ER, PR, and HER2-neu expression. In a recently published validation study, authors reported the prognostic stage provides more accurate prognostic information than does the anatomic stage alone, thus supporting the use of prognostic stage in breast cancer staging.^38^ In the current study, cancer in the majority of patients (82.3%) was detected at an early prognostic stage (0-I), and patients with interval cancers were more likely to have a worse prognostic stage (OR, 3.59; 95% CI, 0.9 to 14.5) with high Ki-67 scores (OR, 3.14; 95% CI, 0.9 to 11.2) compared with screen-detected cancers. Of note, patients with larger tumors, axillary positivity, multifocal/multicentric disease, more-advanced prognostic stage, nonluminal A tumors, and interval cancers were more likely to exhibit worse 5-year DSS and DFS, similar to what has been reported in some studies.^13,35^ The poor prognosis associated with interval cancers has been suggested to be attributed to stage migration, depending on the higher incidence of larger size and axillary positivity. In this study, interestingly, we demonstrated a survival advantage in screen-detected cancers compared with interval cancers among patients with prognostic stage I. However, these findings seem to be contrary to those of O’Brien et al,^39^ who did not demonstrate any survival difference between interval cancers (n = 927) and screen-detected cancers (n = 3,078) after adjusting for some variables, including stage, grade, and tumor subtype.

Similar to other studies,^12,30,33^ we also found that interval cancers more likely presented with larger tumor and with axillary positivity than did screen-detected cancers. In our screening program, the majority of patients with screen-detected tumors chose breast conservation (84%) and SLNB alone (73%). Similarly, in the Sihto et al study,^12^ the surgical approach tended to be more conservative in the screen-detected group compared with women who were symptomatic at diagnosis (88.0 *v* 74.7%; *P* = .005). Furthermore, interval cancers detected within 1 year after a negative screening in our study were more likely to be associated with dense breasts, in concordance with Sala et al,^40^ who also found an association of interval cancers with more-advanced stage, denser breasts, and higher percentages of triple-breast cancers.

We previously demonstrated that our population-based, organized screening project was feasible for women between ages 40 and 49 years in Turkey, with an attendance rate of 88.3% in the first round.^20^ The overall cancer detection rate was 4.8% per 10,000 women, and DCIS, stage I cancer, and axillary-node positivity rates were 22%, 61%, and 17%, respectively. Upon our findings that more than half of the patients had their cancer detected when they were younger than the age of 50 years in the first round in 2011, the Turkish Ministry of Health revised the national cancer-screening standards in 2012, recommending breast cancer screening be initiated at age 40 years instead of age 50 years and biennially up to age 69 years. Under this strategy, cancer in most of our patients was diagnosed at an early anatomic (63.1%) and prognostic (82.3%) stage in the current study. In addition, our screening program also was cost-effective in Turkey, which may be attributed to the early treatment modalities.^41^ Studies are needed to investigate whether our results might be relevant and adaptable to other Asian countries to establish policy recommendations on breast cancer early-detection strategies.^42,43^

In conclusion, our findings suggest that the majority of screen-detected breast cancers mostly exhibit luminal A subtype, in concordance with previous studies from developed countries. However, more aggressive subtypes such as triple-negative cancers are less likely to be detected by mammographic screening programs and may require other imaging modalities, including abbreviated breast MRI.^44^ We are conducting an ongoing trial of abbreviated breast MRI to screen women with dense breasts. New strategies are required for improved screening in developing countries.
